# Bioinformatic analysis predicts that ethanol exposure during early development causes alternative splicing alterations of genes involved in RNA post-transcriptional regulation

**DOI:** 10.1371/journal.pone.0284357

**Published:** 2023-04-13

**Authors:** Camilo Fuentes-Beals, Montserrat Olivares-Costa, María Estela Andrés, Paola A. Haeger, Gonzalo Riadi, Carlos Oliva, Fernando Faunes

**Affiliations:** 1 Ph.D. Program in Sciences Mention Modeling of Chemical and Biological Systems, School of Bioinformatics Engineering, Center for Bioinformatics, Simulation, and Modeling, CBSM, Department of Bioinformatics, Faculty of Engineering, University of Talca, Campus Talca, Talca, Chile; 2 Departamento de Ciencias Biomédicas, Facultad de Medicina, Universidad Católica del Norte, Coquimbo, Chile; 3 Department of Cellular and Molecular Biology, Faculty of Biological Sciences, Pontificia Universidad Católica de Chile, Santiago, Chile; 4 ANID-Millennium Science Initiative Program Millennium Nucleus of Ion Channels-Associated Diseases (MiNICAD), Center for Bioinformatics, Simulation and Modeling, CBSM, Department of Bioinformatics, Faculty of Engineering, University of Talca, Talca, Chile; 5 Departamento de Ciencias Biológicas, Facultad de Ciencias de la Vida, Universidad Andres Bello, Viña del Mar, Chile; Texas Tech Health Sciences Center El Paso, UNITED STATES

## Abstract

Prenatal ethanol exposure is associated with neurodevelopmental defects and long-lasting cognitive deficits, which are grouped as fetal alcohol spectrum disorders (FASD). The molecular mechanisms underlying FASD are incompletely characterized. Alternative splicing, including the insertion of microexons (exons of less than 30 nucleotides in length), is highly prevalent in the nervous system. However, whether ethanol exposure can have acute or chronic deleterious effects in this process is poorly understood. In this work, we used the bioinformatic tools VAST-TOOLS, rMATS, MAJIQ, and MicroExonator to predict alternative splicing events affected by ethanol from available RNA sequencing data. Experimental protocols of ethanol exposure included human cortical tissue development, human embryoid body differentiation, and mouse development. We found common genes with predicted differential alternative splicing using distinct bioinformatic tools in different experimental designs. Notably, Gene Ontology and KEGG analysis revealed that the alternative splicing of genes related to RNA processing and protein synthesis was commonly affected in the different ethanol exposure schemes. In addition, the inclusion of microexons was also affected by ethanol. This bioinformatic analysis provides a reliable list of candidate genes whose splicing is affected by ethanol during nervous system development. Furthermore, our results suggest that ethanol particularly modifies the alternative splicing of genes related to post-transcriptional regulation, which probably affects neuronal proteome complexity and brain function.

## Introduction

Alcohol (ethanol) exposure during pregnancy is the leading environmental cause of mental disability [1]. Neurological, developmental, and behavioral abnormalities derived from Prenatal Alcohol Exposure (PAE) are grouped as Fetal Alcohol Spectrum Disorder (FASD). The FASD phenotype is highly heterogeneous and can include characteristic dysmorphic facial features, growth defects, and behavior alterations such as anxiety, depression, and impaired learning, which can persist to adulthood [2–4]. The prevalence of FASD is estimated to be between 2–5% of school-age children in the United States and 17% in the UK population [5, 6].

The cellular and molecular mechanisms by which ethanol induces neurodevelopmental disabilities have not been completely elucidated. Several animal models reproduce the characteristics of the FASD phenotype [7]. Cellular effects of ethanol in the central nervous system include neuronal apoptosis [8–10] and defects in proliferation, neurogenesis, synaptogenesis, neuronal branching, and circuit formation [11, 12].

Different ethanol exposure schemes during mammalian pregnancy lead to gene expression alterations early in the developing nervous system [13, 14] as well as in the long term [15–18]. Furthermore, transcriptomic analyses of *in vitro* cell differentiation models show changes in gene expression induced by ethanol [19–21]. Interestingly, ethanol modifies the levels of splicing factors, such as *SRSF2* (*Serine and arginine Rich Splicing Factor 2*) and *SRSF11* [13, 14]. SRSF2 and SRSF11 belong to a family of SR proteins characterized by an RNA recognition motif (RRM) and a domain rich in arginine and serine residues (“RS” domain). SR plays multiple roles in gene expression, including control of constitutive and alternative pre-mRNA splicing, mRNA nuclear export, and mRNA translation [22].

Alternative pre-mRNA splicing is the process by which combinations of exons generate different mature mRNAs or splice variants, thereby increasing the transcriptome and proteome complexity thus influencing cellular function [23]. This process is highly prevalent in genes with roles in nervous system development, including neurogenesis, axon guidance, synapse formation, and neurotransmission [24, 25]. Furthermore, a group of small (3–30 nucleotides) alternative exons, called microexons [26], are pervasively included in the nervous system. Defects in the expression of their main regulator, the splicing factor SRRM4, can lead to neurological disorders [27, 28].

Alternative splicing is regulated during physiological processes such as neuronal activity and development [29, 30]. A recent study quantified the presence of exons in mature transcripts in the cerebral cortex at 9 developmental time points from embryonic day E14.5 to 21 months after birth in mice [30]. The authors found 2,883 exons with developmental changes. Furthermore, distinct biological categories of genes containing these exons with switches at specific developmental windows were identified. For example, genes with exons that begin to be included early (embryonic) were related to ion channels and transmembrane transport. In contrast, genes with exons that start inclusion postnatally were related to cytoskeleton remodeling and synaptogenesis. These results suggest that alternative splicing is highly regulated during nervous system development and that genes belonging to the same biological category can be regulated at specific developmental time points.

Several lines of evidence show that ethanol exposure alters the splicing of some genes in different mammalian species, including humans [31–36]. Whether the effect of ethanol on splicing is nonspecific or specific for a group of genes is an open question. The availability of RNA sequencing (RNA-seq) libraries and bioinformatics tools to predict changes in alternative splicing allows us to address this question.

To study whether alternative splicing is affected by ethanol exposure in the short and long term, we analyzed published RNA-seq libraries using the bioinformatics tools Vertebrate Alternative Splicing and Transcription Tools (VAST-TOOLS) [37], Replicate Multivariate Analysis of Transcript Splicing (rMATS) [38], and Modeling Alternative Junction Inclusion Quantification (MAJIQ) [39]. We selected these tools based on the analysis of the performance to detect changes in alternative splicing between RNA-seq libraries [40], and because they are actively supported and kept up to date. VAST-TOOLS works with VastDB and Matt [41] for downstream analysis of alternative splicing. VastDB is composed of quantitative profiles that describe the inclusion levels and functional associations of alternative splicing events detected in RNA-seq data of vertebrates and tissue types. rMATS applies a statistical method to detect differential alternative splicing events between replicate RNA-seq data. MAJIQ detects Local Splicing Variations (LSVs), defined as splits (multiple edges) in a splice graph. These edges come into or from a single exon, termed reference exon. MAJIQ proposes the LSV methodology to address different shortcomings of defined alternative splicing events. In addition to these three tools, MicroExonator [42] was included for microexon prediction and analysis. Using these approaches, we found genes with predicted differential alternative splicing common to the distinct bioinformatics tools and experimental schemes. In addition, the alternative splicing of genes related to RNA processing and protein synthesis was commonly affected in these different schemes of ethanol exposure.

## Results

**Fig 1:**
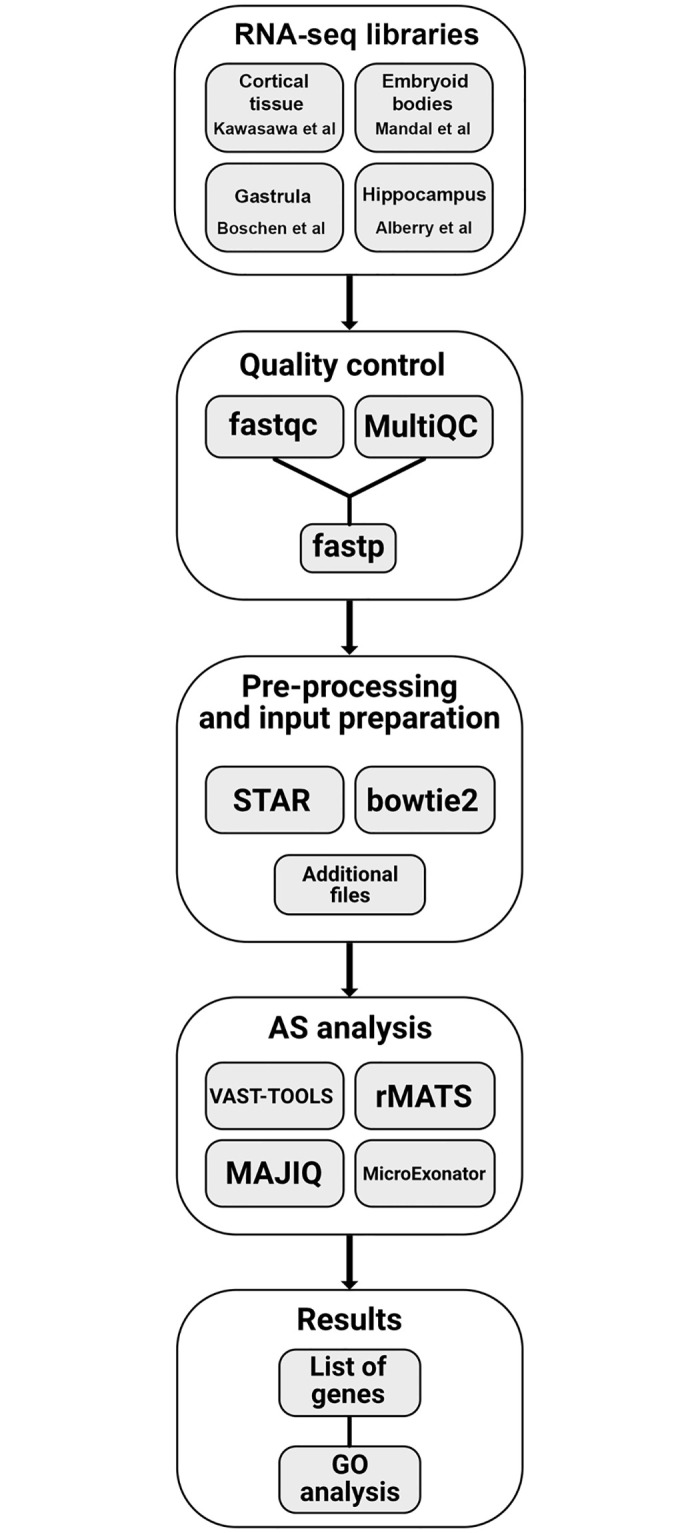
Flow diagram of the data processing. The diagram starts with the four analyzed RNA-seq libraries depicted in the first step, associated with the names of their authors. The second step ‘Quality Control’ shows the software employed for quality check and filtering of the library reads. The output of this step is used as input for ‘Pre-processing and input preparation’, which includes two main software programs required for the Sequence Alignment Map (SAM) file preparation and other additional file requirements. These files are the input for the selected alternative splicing (AS) tools. The ‘AS analysis’ step contains the execution of the selected AS software. Lastly, the ‘Results’ step includes the Gene Ontology (GO) analysis of the genes that show an AS event.

To understand the molecular mechanism underlying the effect of PAE, we focused on alternative splicing as a central point of development. We hypothesized that biological processes affected by ethanol can be determined from the comparison of differential splicing events detected in different experimental schemes using bioinformatics tools. Available RNA-seq data from control and ethanol exposure conditions (ranging from 7 million to 63 million reads per library) were divided into short-term and long-term ethanol effects (Table 1). We selected the freely available bioinformatics tools VAST-TOOLS [37], rMATS [38], MAJIQ [39], and MicroExonator [42] (Fig 1). As a first approach, VAST-TOOLS, rMATS, and MAJIQ were used to find genes with all types of splicing events altered by ethanol.

**Table 1:** Description of RNA-seq libraries analyzed.

Effect	Species	Experimental conditions	Libraries	Reference, GSE/SRA
Short-term	Human	15–18 gestational weak, human cortical slices cultured in a medium containing 50-mM ethanol or PBS (control) for 24 h	Four libraries (2 control, 2 ethanol)	Kawasawa et al 2017
			35-60M single-end/library reads after filtering. 50 a 65 pb	GSE86006
				PRJNA339987
Short-term	Human	Human embryonic carcinoma cells (NCCIT) cultured to form embryoid bodies and treated in the presence and absence of ethanol (50 mM) for 48 h.	Four libraries (2 EB, 2 EB+EtOH)	Mandal et al 2015 [21]
			7-11M paired-end reads/library after filtering. 101 bp	SRP060284
Short-term	Mouse	Embryos E7.0, 12 h after the first alcohol injection (E7.5)	Eleven libraries E7.5 (J strain)	Boschen et al 2021
			(5 control and 6 EtOH)	GSE163796
			52-63M paired-end reads/library. 50 bp	SRP299170
Long-term	Mouse	Samples from hippocampus p70 of mice born from mothers treated with ethanol before and during pregnancy	Six libraries (3 control and 3 EtOH)	Alberry et al 2020 [15]
			17-24M paired-end reads/library	GSE137984
			after filtering. 135–147 bp	SRP223245

Description of the experimental conditions of RNA-seq libraries analyzed in this work.

### Ethanol affects alternative splicing of genes related to RNA processing and chromatin remodeling in the human embryonic cortex

**Fig 2:**
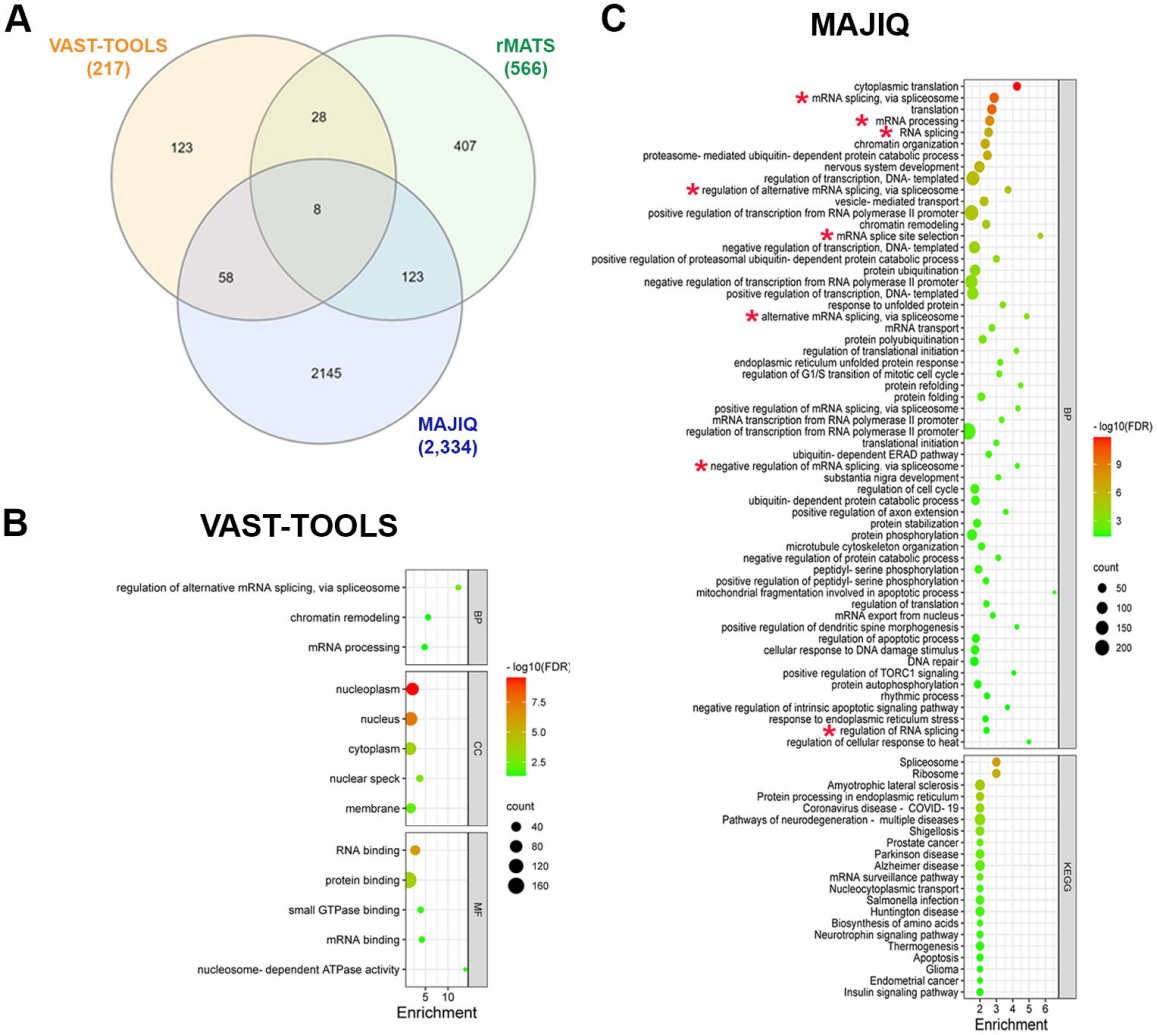
Analysis of alternative splicing events predicted to be altered by ethanol in the human embryonic cortical tissue dataset. **A)** Venn diagram showing the number of genes predicted to undergo altered alternative splicing events due to ethanol exposure, using VAST-TOOLS, rMATS, and MAJIQ. **B, C)** Gene Ontology analysis of genes predicted to contain alternative splicing events altered by ethanol exposure, using VAST-TOOLS **(B)** and MAJIQ **(C)**. BP, Biological Process, CC, Cellular Component, MF, Molecular Function, KEGG, Kyoto Encyclopedia of Genes and Genomes, FDR, False Discovery Rate.

We used data from RNA-seq libraries prepared from human cortical tissue isolated from gestational weeks 15–18 exposed to 50 mM ethanol for 24 hours [20]. Our comparison between control and ethanol libraries showed that 235 differential alternative splicing events (from 217 genes) were predicted with VAST-TOOLS, 702 events (from 566 genes) were predicted with rMATS, and 3,807 events (from 2,334 genes) were predicted with MAJIQ (S1 Table). The intersection of these lists showed 8 common genes to all lists and 209 genes common to at least two lists (Fig 2A).

Gene Ontology (GO) analysis of genes with alternative splicing events altered by ethanol exposure predicted by VAST-TOOLS showed enrichment in genes related to “regulation of alternative splicing”, “chromatin remodeling”, and “mRNA processing” (Fig 2B and S1 Table). Consistently, the cellular components “nucleoplasm”, “nucleus”, and “nuclear speck”, and the molecular function “RNA binding” were also enriched (Fig 2B).

MAJIQ predicted 2,334 genes with alternative splicing events altered by ethanol in the human embryonic cortical tissue dataset. Among these genes, 58 Biological Processes categories were enriched and 8 out of these 58 categories were related to mRNA splicing and processing (red asterisks in Fig 2C and S1 Table). Several categories related to translation were also enriched in the MAJIQ-predicted genes. “Nucleus” was the most enriched cellular component category and “Protein binding”, “RNA binding”, and “mRNA binding” were the top enriched Molecular Function categories (S1A Fig). Importantly, the KEGG Pathways “Spliceosome” and “Ribosome” were the top enriched categories (Fig 2C). These results suggest that the alternative splicing of genes related to post-transcriptional processes was significantly altered by ethanol in the cortex. In addition, this analysis showed that, even when the number of shared genes predicted by these two bioinformatics tools was low, common biological processes and pathways can be identified from the list of genes.

In contrast to the results obtained with VAST-TOOLS and MAJIQ, rMATS predicted only differential alternative splicing events of genes related to molecular functions “protein binding”, “kinase activity”, and “ATP binding” (S1B Fig and S1 Table). No biological processes and KEGG pathways were enriched in the set of genes predicted by rMATS.

**Fig 3:**
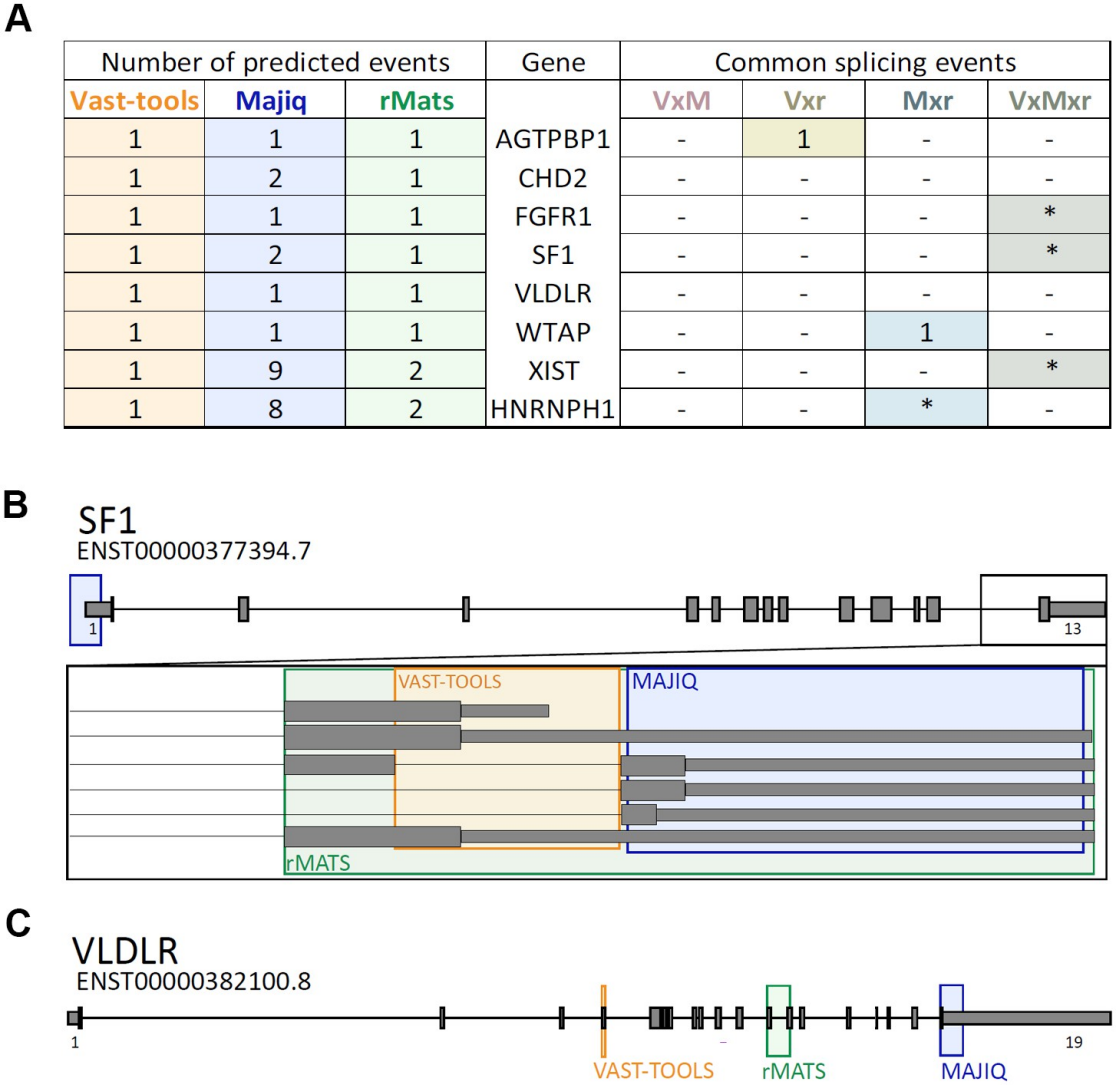
Analysis of genes with alternative splicing events predicted to be altered by ethanol in the human embryonic cortical tissue dataset. **A)** Number of alternative splicing events predicted for the 8 common genes by each bioinformatics tool (left side of the table). The number of splicing events predicted for at least two bioinformatics approaches is shown on the right side of the table. * Indicates that the alternative splicing events were not located at the same genomic coordinates, but they are contiguous or overlapped as shown in B. Comparison between bioinformatics predictions are coded as follow: VxM; VAST-TOOLS and MAJIQ matches, Vxr; VAST-TOOLS and rMATS matches, Mxr; MAJIQ and rMATS matches, VxMxr; VAST-TOOLS, MAJIQ, and rMATS matches. **B)** Diagram of the SF1 gene (ENST00000449182.1) illustrating the location of predicted splicing events. The lower part of the diagram represents a magnification of the 3´ end of the gene. **C)** Diagram of the VLDLR gene (ENST00000382100.8) illustrating the location of predicted splicing events. Blue boxes represent the coordinates of MAJIQ predicted events. Green boxes represent the coordinates of rMATS predicted events. Yellow boxes represent the coordinates of VAST-TOOLS predicted events. Intron sequences are represented by lines, exons by grey boxes, and UTR sequences by slim grey boxes. Gene diagrams are represented from 5´to 3´. Small numbers under the first and last exons indicate the number of the corresponding exon according to GENCODE V41.

A detailed analysis of the 8 common genes predicted by VAST-TOOLS, MAJIQ, and rMATS showed that distinct alternative splicing events differentially altered by ethanol were detected by one or another bioinformatics tool (Fig 3A). Strikingly, some predicted splicing events were not located at the same genomic coordinates but were adjacent or overlapped, suggesting that ethanol-induced splicing alterations occur in a specific gene region. For example, the three bioinformatics tools predicted changed ethanol-induced splicing events in the last exons of the SF1 gene, including the coding sequences and the 3’UTR. However, each bioinformatics tool predicted different splicing events within this region of the SF1 gene (Fig 3B). Furthermore, other genes such as *VLDLR*, *CHD2*, *XIST*, and *HNRNPH1* underwent ethanol-induced changes in multiple of the predicted gene-wide splicing events (Fig 3C). Remarkably, 4 genes (*CHD2*, *HNRNPH1*, *SF1*, *WTAP*) out of these 8 common genes play roles in splicing regulation, supporting the idea that ethanol may affect the control of the splicing process.

### Ethanol modifies alternative splicing of RNA binding-related genes during the differentiation of human embryoid bodies

**Fig 4:**
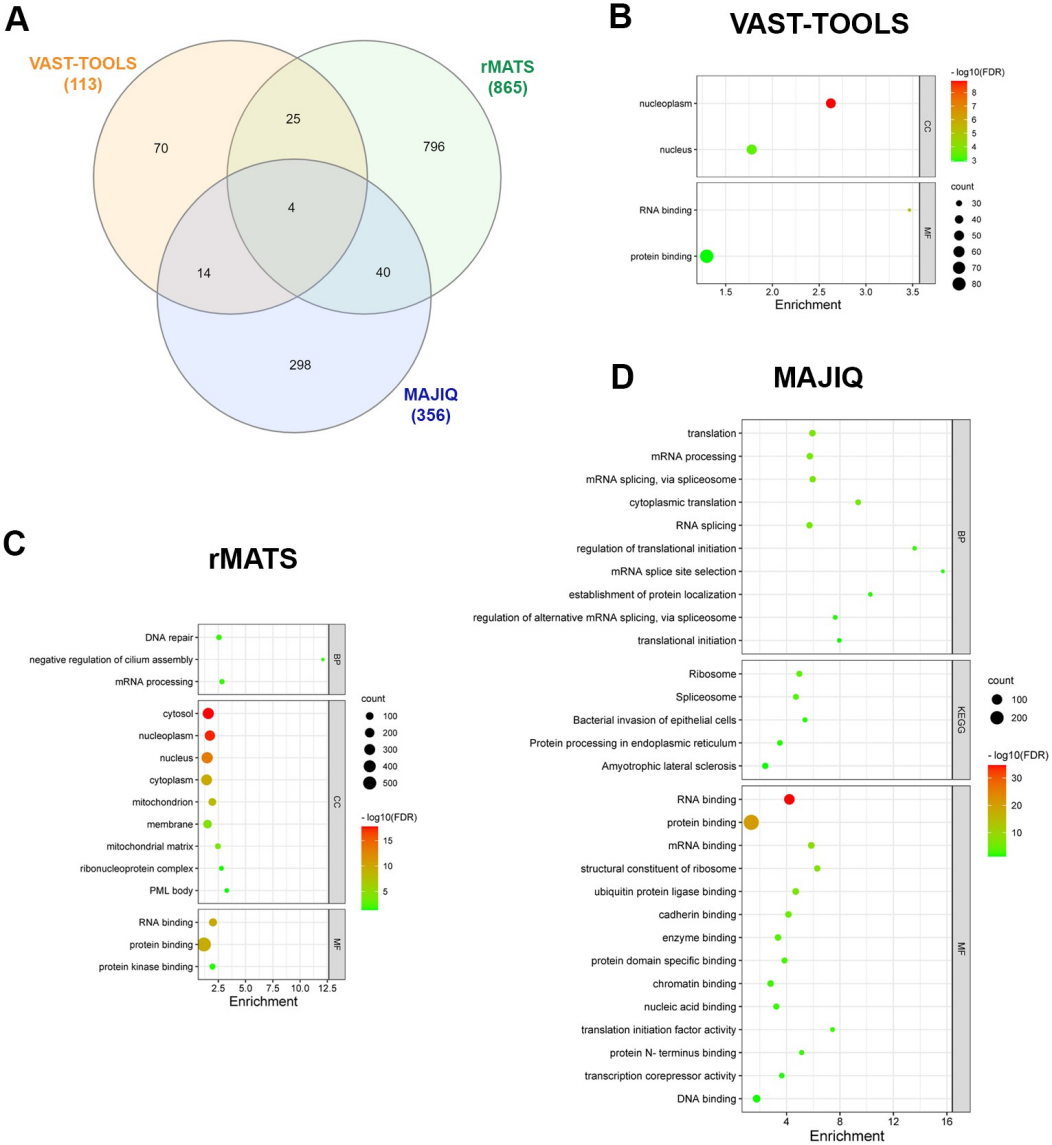
Analysis of alternative splicing events predicted to be altered by ethanol in the human embryoid bodies differentiation dataset. **A)** Venn diagram showing the number of genes predicted to contain alternative splicing events altered by ethanol using VAST-TOOLS, rMATS, and MAJIQ. **B—D)** Gene Ontology analysis of genes predicted to contain alternative splicing events altered by ethanol by VAST-TOOLS **(B)**, rMATS **(C)**, and MAJIQ **(D)**. BP, Biological Process, CC, Cellular Component, MF, Molecular Function, KEGG, Kyoto Encyclopedia of Genes and Genomes, FDR, False Discovery Rate.

To study the effect of ethanol on early steps of differentiation, we analyzed the libraries prepared from human embryonic carcinoma cells, cultured to form embryoid bodies in the absence and presence of ethanol for 48 hours [21]. VAST-TOOLS predicted 122 differential alternative splicing events (from 113 genes), MAJIQ predicted 537 events (from 356 genes), and rMATS predicted 1100 events (from 865 genes) (S2 Table). The intersection of the findings from these three tools revealed 4 common genes in the three lists and 79 genes predicted by at least two bioinformatics tools (Fig 4A).

GO analysis of gene datasets with ethanol-induced altered splicing in embryoid bodies showed an enrichment of the molecular function “RNA binding” with the three bioinformatics tools (Fig 4B–4D and S2 Table). The biological process “mRNA processing” was also enriched in rMATS and MAJIQ lists. MAJIQ list of genes showed enrichment in cell processes such as “translation”, “RNA splicing”, “mRNA splicing, via spliceosome”, “cytoplasmic translation”, and “regulation of alternative mRNA splicing, via spliceosome”, and the KEGG pathways “Ribosome” and “Spliceosome” (S2 Table).

**Fig 5:**
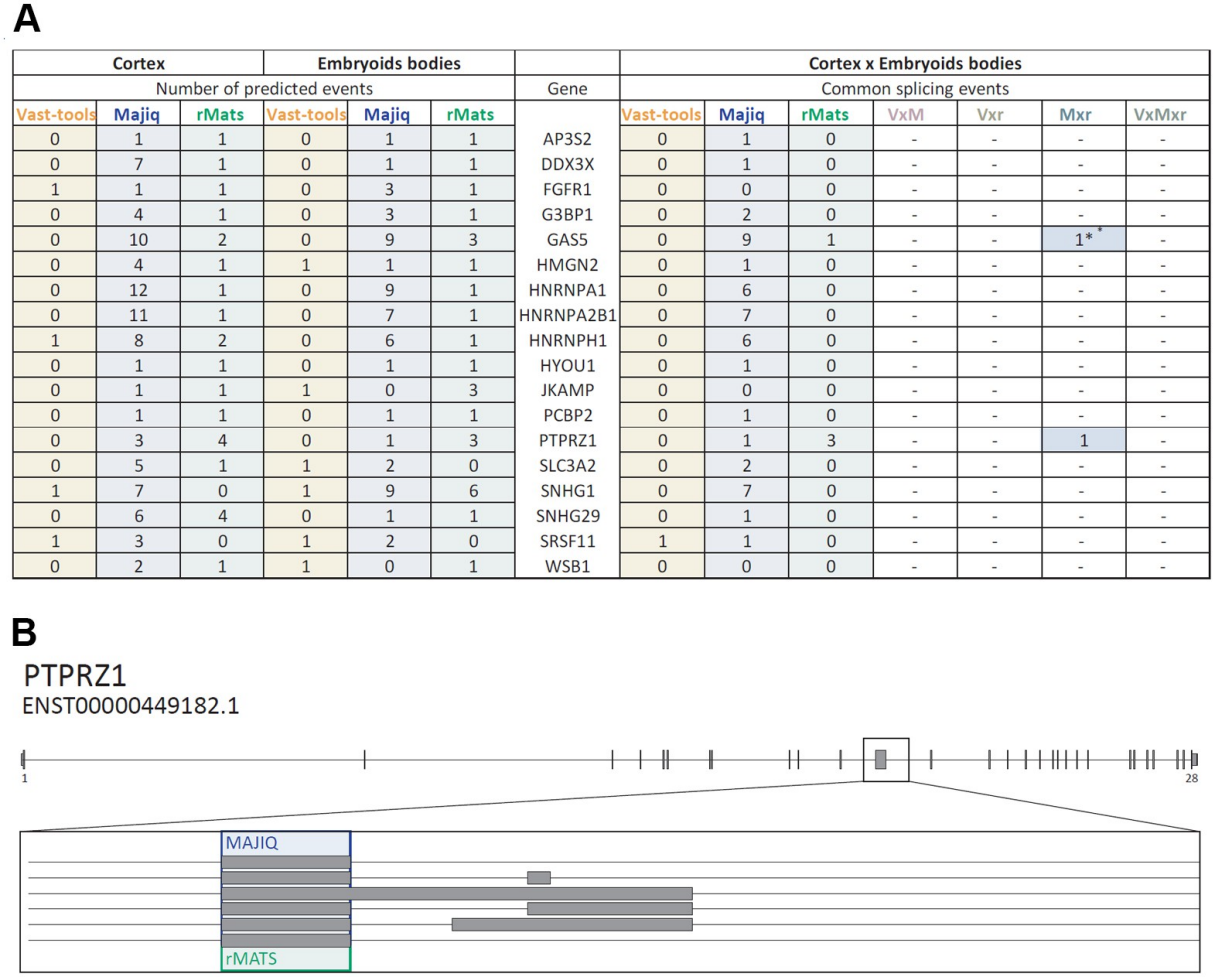
Common splice events predicted to be altered by ethanol in the cortex and embryoid bodies datasets. **A)** Common genes whose alternative splicing was predicted to be affected by ethanol in the cerebral cortex and embryoid bodies. The left side of the table details the number of splicing events predicted by each of the bioinformatics tools. The right side of the table shows the number of splicing events common for cerebral cortex and embryoid bodies predicted by each of the bioinformatics tools, and the number of splicing events common for cerebral cortex and embryoid bodies predicted by at least two of them. * Indicates that the alternative splicing events do not have the same coordinates, but they overlap. Comparison between bioinformatics predictions are coded as follows: VxM; VAST-TOOLS and MAJIQ matches, Vxr; VAST-TOOLS and rMATS matches, Mxr; MAJIQ and rMATS matches, VxMxr; Vast-tools, MAJIQ, and rMATS matches. **B)** Diagram of the BPTPBZ1 gene (ENST00000449182.1) illustrating the location of the splicing event predicted to be affected by ethanol in the cerebral cortex and embryoid bodies according to MAJIQ and rMATS analyses. The blue box represents the coordinates of MAJIQ predicted event and the green box represents the coordinates of rMATS predicted event. Intron sequences are represented by lines, exons by grey boxes, and UTR sequences by slim grey boxes. The diagrams show the genes in the 5´to 3´ direction. Small numbers under the first and last exons indicate the number of the corresponding exon according to GENCODE V41.

To identify common biological processes altered by ethanol both during nervous system development and differentiation of all germinal layers, we compared the 83 genes of the embryoid bodies differentiation dataset and the 217 genes of the embryonic cortical tissue dataset to look for common genes with differential alternative splicing predicted with at least two bioinformatics tools. This analysis revealed 18 common genes whose alternative splicing was predicted to be affected by ethanol (*AP3S2*, *DDX3X*, *FGFR1*, *G3BP1*, *GAS5*, *HMGN2*, *HNRNPA1*, *HNRNPA2B1*, *HNRNPH1*, *HYOU1*, *JKAMP*, *PCBP2*, *PTPRZ1*, *SLC3A2*, *SNHG1*, *SNHG29*, *SRSF11*, *WSB1*). Among these genes, we found several shared splicing events, mainly predicted by MAJIQ or rMATS (Fig 5). Interestingly, *GAS5* and *PTPRZ1* possess a splicing event altered by ethanol predicted by MAJIQ and rMATS in both datasets (Fig 5).

Notably, this group of 18 genes was significantly enriched in the molecular functions “RNA binding” (9 genes), “miRNA binding” (3 genes), and “nucleic acid binding” (4 genes), and in the cellular component “ribonucleoprotein complex” (5 genes) (S3 Table). Therefore, genes whose alternative splicing was modified by ethanol were enriched in RNA processing and translation, suggesting that ethanol affects gene expression through post-transcriptional mechanisms during the early steps of differentiation. In addition, these findings also indicate that the impact of ethanol exposure in these categories is not limited to the nervous system.

### Ethanol alters the alternative splicing of genes related to RNA processing during germ layer specification in mice

**Fig 6:**
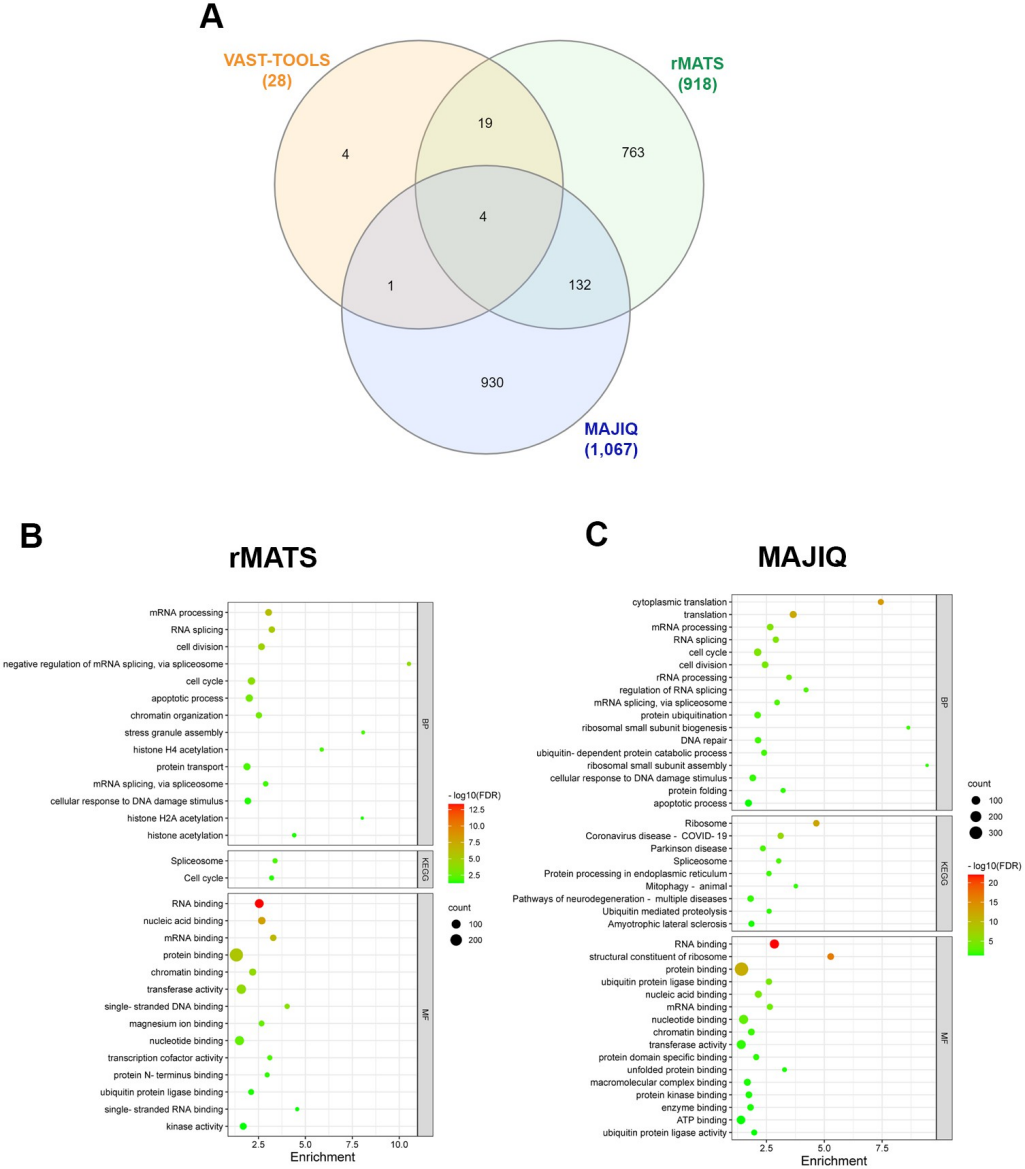
Analysis of alternative splicing events predicted to be altered by ethanol in the mouse gastrula dataset. **A)** Venn diagram showing the number of genes predicted to undergo alternative splicing events altered by ethanol using VAST-TOOLS, rMATS, and MAJIQ. **B, C)** Gene Ontology analysis of genes predicted by rMATS **(B)** and MAJIQ **(C)** to undergo alternative splicing events altered by ethanol. BP, Biological Process, MF, Molecular Function, KEGG, Kyoto Encyclopedia of Genes and Genomes, FDR, False Discovery Rate.

To further explore the effects of ethanol on alternative splicing, we analyzed RNA-seq libraries prepared from whole gastrula embryos after 12 hours of ethanol exposure by intraperitoneal injection of pregnant mice (from stage E7.0 or E7.5, embryonic day 7) [13]. VAST-TOOLS predicted 28 differential alternative splicing events (from 28 genes), MAJIQ predicted 1312 events (from 1067 genes), and rMATS predicted 1,308 events (from 918 genes) (S4 Table). The intersection of these lists revealed 4 common genes detected by the three tools and 152 common to at least two (Fig 6A).

Very similar to results obtained from the ethanol-exposed human embryonic cortical tissue and embryoid bodies differentiation datasets, MAJIQ and rMATS predicted ethanol-induced differential alternative splicing of genes enriched in several categories related to RNA metabolism such as “RNA splicing”, “RNA binding”, and “spliceosome” and to protein synthesis such as “translation” and “Ribosome” (Fig 6B and 6C, S3 Fig and S4 Table). VAST-TOOLS predicted 28 events, but no biological processes or molecular function categories were enriched. In summary, ethanol affects the alternative splicing of genes related to RNA processing and translation in human embryonic cortical tissue, human embryoid bodies, and mouse gastrula, suggesting a common effect in post-transcriptional processes during early differentiation steps that is conserved in mice and humans.

### Long-term effect of ethanol on the mice brain

To compare the early and long-term effects of ethanol exposure on the nervous system, we analyzed libraries published by Alberry et al [15]. Adult female mice were exposed to ethanol for 10 days and then they were mated. Pregnant females were exposed to ethanol until postnatal day 10 and RNA-seq libraries were prepared from the hippocampus of the litter at postnatal day 70 (P70). In this dataset, VAST-TOOLS predicted no differential alternative splicing events, rMATS predicted 1 event, and MAJIQ predicted 22 events (from 9 genes) (S5 Table). No GO categories or KEGG pathways were enriched in these lists. Although this is a different experimental approach than the short-term ethanol treatments discussed above, this result suggests that in the long-term no specific process related to alternative splicing is affected by ethanol.

### Microexon inclusion is altered by ethanol

Microexons are small exons (< 30 nucleotides) that are pervasively included during the development of the nervous system. Microexons require specific factors for their splicing. Misregulation of microexon inclusion results in neurodevelopmental disorders such as autistic spectrum disorder (ASD) [27]. Since ASD and FASD are neurodevelopmental in origin and their symptoms overlap, learning whether ethanol alters microexon inclusion is of utmost interest. To this end, we used MicroExonator [42] to study whether ethanol altered the microexon inclusion rate.

MicroExonator predicted 118 ethanol-induced differential microexon inclusion events (from 104 genes) in the human embryonic cortical tissue dataset, 12 events in the embryoid bodies differentiation dataset (from 11 genes), and 19 events (from 19 genes) in the mouse gastrula dataset (S1, S2 and S4 Tables). No differential microexon inclusion events were predicted in the long-term effect dataset. The intersection of the three lists of genes with differential microexon inclusion showed no genes common to the three lists. However, three genes were common to embryonic cortical tissue and embryoid bodies lists (*CLASP2*, *DOCK7*, and *RPS24*) and the same inclusion of microexons for *DOCK7* and *RPS24* were predicted to be altered by ethanol. Using MicroExonator, we found 4 differential inclusion events predicted for *PTPRD1* and two for *SHANK2* in the human embryonic cortical tissue dataset. Although both genes were previously predicted as ethanol-induced altered microexon inclusion in the original paper, there is a partial overlap of the microexons found between our prediction and that made in the original work [20].

## Discussion

A substantial body of literature has shown the effect of ethanol on gene expression [13, 15, 21, 43]. However, much less attention has been paid to the effect of ethanol on alternative splicing [20, 36, 44]. For a critical analysis of the effect of ethanol, it is important to consider the administration window and the timing of the assessment of the treatment effect. In this work, we classified available datasets into those that assess short-term and long-term changes. Using these datasets and experimentally validated bioinformatics tools, we found that ethanol exposure during development or early steps of differentiation *in vitro* does change the pattern of alternative splicing in the short term. Considering the myriad of cellular processes that require a proper alternative splicing regulation during the development of tissues and organs [23], it is not surprising that alterations in splicing, even for a limited time, can have permanent deleterious effects in the structure and physiology of the nervous system and other tissues. Interestingly, in the only dataset analyzed that was classified as long-term assessment [15], we found few significant changes in alternative splicing, precluding the obtainment of enriched GO terms. In this FASD model, consisting of chronic ethanol treatment (starting before mating up to postnatal day 10) and hippocampal samples isolated on postnatal day 70, ethanol could influence the functioning of several complementary mechanisms that massively affect brain development. These mechanisms may mask the early developmental splicing process as well as the maintenance of proteomic variability in postnatal development. Nevertheless, the prediction of changes in alternative spicing after short exposures to ethanol supports the notion that these transient alterations may result in permanent changes in the organism.

Interestingly, even with the scarce overlap of genes and alternative splicing events among VAST-TOOLS, rMATS, and MAJIQ, we found common cellular GO processes significantly enriched in genes with differential alternative splicing altered by ethanol when comparing independent datasets with different experimental approaches. These results suggest a specific and conserved effect of ethanol during the early steps of differentiation and development of the nervous system. Importantly, we found an overrepresentation of genes classified as RNA regulators, such as splicing and RNA binding proteins. Thus, our results indicate that RNA regulation is one of the targets of ethanol during development. The misregulation of alternative splicing in RNA regulators may result in the generation of new protein domains with different interacting partners, the generation of truncated proteins, or the activation of nonsense-mediated decay. The specific effect in candidates identified here requires further exploration, highlighting the relevance of studying alternative splicing isoforms in addition to mRNA levels to understand the molecular mechanisms underlying FASD.

Because the specific sensitivity and selectivity of each bioinformatics tool are important parameters in these bioinformatics analyses, the use of several software programs is highly recommended to predict differential alternative splicing between two conditions. For example, *PTPRD1* and *SHANK2* alternative splicing events of microexons altered by ethanol, which were validated by RT-qPCR [20], were not predicted by VAST-TOOLS, rMATS, and MAJIQ in the same dataset of human cortical tissue. Only Microexonator predicted one of the events of *SHANK2*, suggesting that different approaches are useful to predict differential alternative splicing events. In addition, the depth of sequencing and the number of replicates may also impact the number of events and genes predicted using these tools. In summary, this bioinformatics analysis provides a list of candidate genes whose splicing is affected by ethanol during nervous system development and suggests that alternative splicing of genes involved in post-transcriptional regulation is altered by ethanol exposure.

## Materials and methods

### RNA-seq libraries and pre-processing

An exhaustive search of all publicly available RNA-seq libraries related to ethanol exposure on development and differentiation of the nervous system was done in NCBI’s Sequence Read Archive (SRA) [45]. The 4 selected RNA-seq libraries are described in Table 1. The quality of each RNA-seq library was analyzed with FastQC v0.11.9 [46] and the output was processed with MultiQC [47]. Trimming and deduplication were done using fastp [48] with default parameters plus *‘—dedup’*.

### Genome and annotation files

For VAST-TOOLS, the genomes of *Homo sapiens* (hg38), and *Mus musculus* (mm10) (Ensembl v88) were downloaded. The genomes and annotations files used in rMATS, MAJIQ, and MicroExonator were downloaded from the UCSC genome browser (https://hgdownload.soe.ucsc.edu/downloads.html) and GENCODE in *gff3* and *gtf* formats (https://www.gencodegenes.org) (*Homo sapiens* GRCh38 p13 with GENCODE annotation GRCh38.p13, *Mus musculus* GRCm38 p6 with GENCODE annotation GRCm38.p5).

### Prediction of alternative splicing events altered by ethanol

Input files were prepared according to the README instructions for each tool. rMATS requires the mapping of the reads using the STAR aligner [49] (v2.7.10a). MAJIQ requires a sorted BAM file (Binary Sequence Alignment Mapping of reads) as input. This was done using bowtie2 v2.4.5 [50]. Both aligners were run with default parameters. MicroExonator uses *gtf* (https://www.gencodegenes.org/pages/data_format.html) gene transfer format (gtf)), and *bed12* (https://bedtools.readthedocs.io/en/latest/content/general-usage.html) browser extensible data (bed), formats for annotation files. The *bed12* files refer to a transcript annotation file obtainable from the UCSC table browser (https://genome.ucsc.edu/cgi-bin/hgTables). Also, a *bigWig* file containing genome-wide conservation scores of related organisms is required. For selected species, this file is available from the UCSC genome browser (http://hgdownload.cse.ucsc.edu/downloads.html). The detailed execution of each software is provided in S1 Appendix.

Here, we briefly describe how each tool predicts alternative splicing events and compares experimental conditions. VAST-TOOL, rMATS, and MAJIQ detect Exon skipping, mutually exclusive exons, Alternative 5’ donor site, Alternative 3’ receptor site, and Intron retention. VAST-TOOL and MicroExonator also detect microexons. VAST-TOOL uses EST, cDNA evidence, gene annotations, and evolutionary conservation to assemble libraries of Exon-Exon Junctions (EEJ). These libraries are used in the detection and quantification of alternative splicing sequences from RNA-seq reads. The exon inclusion level is determined by PSI metric (or percent spliced in). Quantifications are based on read counts corrected for the number of mappable positions in each EEJ. In this work, we used the default ΔPSI> = 15 to consider an event with differential inclusion between control and ethanol libraries. MAJIQ parses a known database of transcripts (usually a GFF3 file), along with a set of mapped and aligned RNA-seq experiments. As MAJIQ works with LSV, several filters can be applied to define the edges of the LSV and assess its reliability. The quantification process is based on the marginal percent index (PSI) for each junction involved in the LSV. A combination of read rate modeling, Bayesian PSI modeling, and bootstrapping is used to report posterior PSI and ΔPSI distributions for each quantified LSV. Here, we used the default values for ΔPSI, ranging from -1 to 1 to consider an event with differential inclusion between control and ethanol libraries. This range represents the posterior distribution of the quantification data. In rMATS, the reads mapped to different isoforms are used to estimate their proportions. For quantification, the exon inclusion level (PSI) is estimated by the count of reads specific to the exon inclusion isoform and the exon skipping isoform. A binomial distribution is assumed for the inclusion read counts, allowing us to model the estimation uncertainty of PSI and we used the default threshold for ΔPSI > = 5% to consider the difference between the samples. MicroExonator carries a *de-novo* search for unannotated microexons and subsequently quantifies both new and previously annotated microexons. Alignment of the RNA-seq reads with different program options gives the basis for the detection and quantification of microexons. Only microexons larger or equal to 8 nucleotides that map to exon-exon junction coordinates are counted. This putative list is preprocessed for quantification and filtered according to program parameters, and a score is used to determine whether the microexon is from a real splicing event or a spurious match. In this work, we used the default ΔPSI > = 0.1 to consider an event with differential inclusion between control and ethanol libraries. This considers a score obtained from fitting the quantification data to a specific distribution.

### Gene ontology and KEGG pathways analysis

Official names of genes were loaded into the DAVID platform (version Dec. 2021) and the complete set of genes of the species was used as background (default option) (Dennis et al., 2003). Functional Annotation Tool was used, and the Gene Ontology options GOTERM_BP_DIRECT, GOTERM_CC_DIRECT, GOTERM_MF_DIRECT, and KEGG_PATHWAY were selected. For analysis, only GO terms and KEGG pathways with FDR < 0.05 were considered. Results were exported and plotted using SRplot (http://www.bioinformatics.com.cn/plot_basic_gopathway_enrichment_bubbleplot_081_en).

### Visualization of alternative splicing events

Predicted splicing events were mapped in the *Homo sapiens* (hg38) genome using the genome browser tool of the University of California Santa Cruz (UCSC) [51]. Alternative splicing events were defined as common between two or more bioinformatics approaches when they share the same position in the genome. Partially overlapped, or adjacent events were considered different splicing occurrences.

## Supporting information

S1 Fig Analysis of alternative splicing events predicted to be altered by ethanol in the human embryonic cortical tissue dataset. Gene Ontology analysis of genes predicted to contain alternative splicing events altered by ethanol by MAJIQ **(A)** and rMATS **(B)**. CC, Cellular Component, MF, Molecular Function, FDR, False Discovery Rate.
(TIF)

S2 Fig Analysis of alternative splicing events predicted to be altered by ethanol in the human embryoid bodies differentiation dataset. Gene Ontology analysis of genes predicted to contain alternative splicing events altered by ethanol by MAJIQ. CC, Cellular Component, FDR, False Discovery Rate.
(TIF)

S3 Fig Analysis of alternative splicing events predicted to be altered by ethanol in the mouse gastrula dataset. Gene Ontology analysis of genes predicted to contain alternative splicing events altered by ethanol by MAJIQ. CC, Cellular Component, FDR, False Discovery Rate.
(TIF)

S1 Table Alternative splicing events predicted to be altered by ethanol in the human embryonic cortical tissue dataset. Datasheets including VAST-TOOLS, rMATS, MAJIQ, Microexonator and Gene Ontology results.
(XLSX)

S2 Table Alternative splicing events predicted to be altered by ethanol in the human embryoid bodies differentiation dataset. Datasheets including VAST-TOOLS, rMATS, MAJIQ, Microexonator and Gene Ontology results.
(XLSX)

S3 Table Gene Ontology analysis of genes with common splice events predicted to be altered by ethanol in the cortex and embryoid bodies datasets. Gene Ontology results of genes predicted to be altered by ethanol in the cortex and embryoid bodies datasets.
(XLSX)

S4 Table Alternative splicing events predicted to be altered by ethanol in the mouse gastrula dataset. Datasheets including VAST-TOOLS, rMATS, MAJIQ, Microexonator and Gene Ontology results.
(XLSX)

S5 Table Alternative splicing events predicted to be altered by ethanol in the mice brain. Datasheets including VAST-TOOLS, rMATS, MAJIQ, Microexonator and Gene Ontology results.
(XLSX)

S1 Appendix Detailed execution of alternative splicing software. Description of the execution of Alternative Splicing Software used in this work.
(DOCX)
