# Supplementary figures and images for: Bioinformatic analysis predicts that ethanol exposure during early development causes alternative splicing alterations of genes involved in RNA post-transcriptional regulation
Source: PLoS One. 2023 Apr 13;18(4):e0284357. doi: 10.1371/journal.pone.0284357 (PMC10101408; doi:10.1371/journal.pone.0284357)

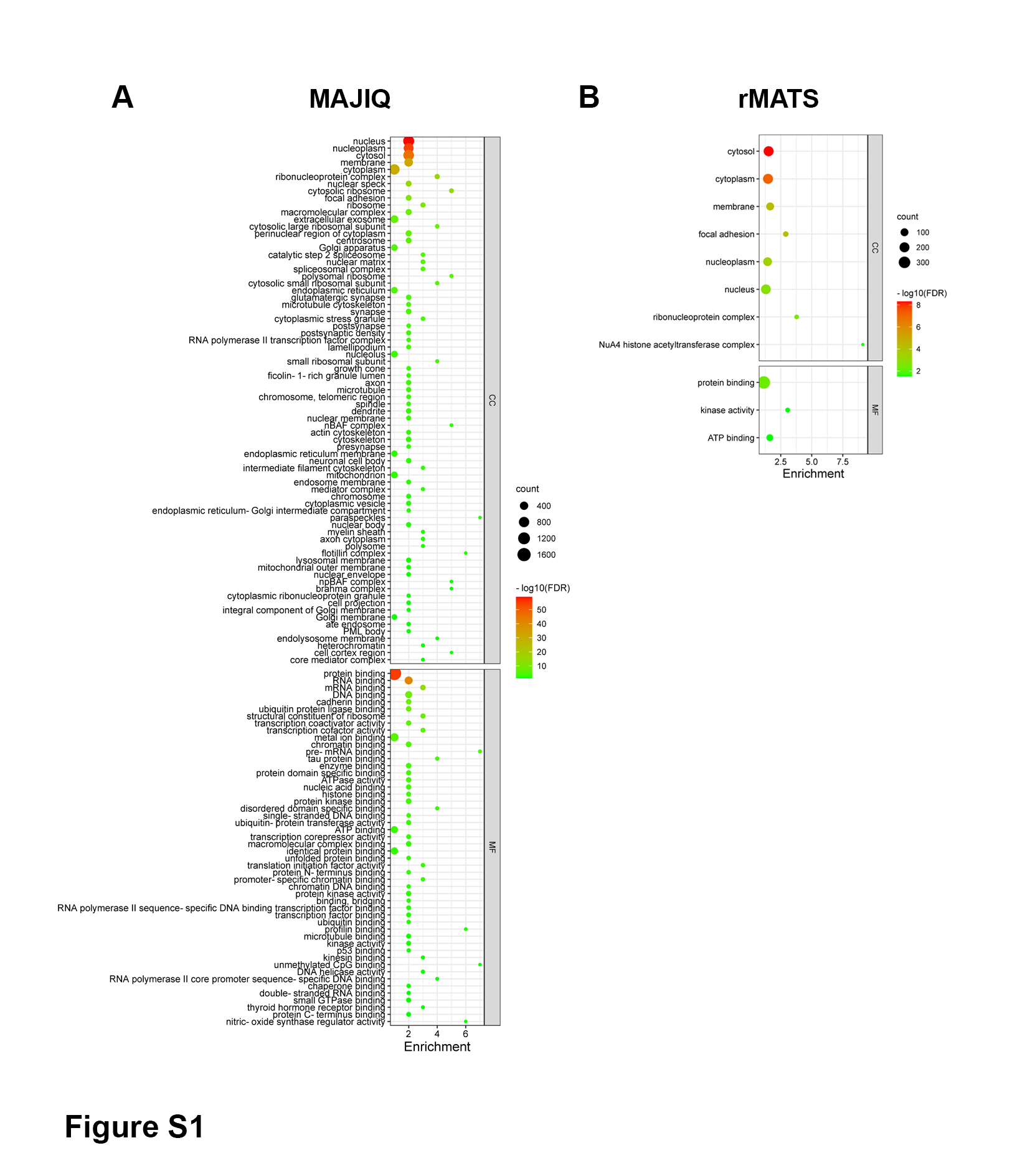

Supplement: S1 Fig — Gene Ontology analysis of genes predicted to contain alternative splicing events altered by ethanol by MAJIQ (A) and rMATS (B). CC, Cellular Component, MF, Molecular Function, FDR, False Discovery Rate. (TIF) [file pone.0284357.s001.tif]

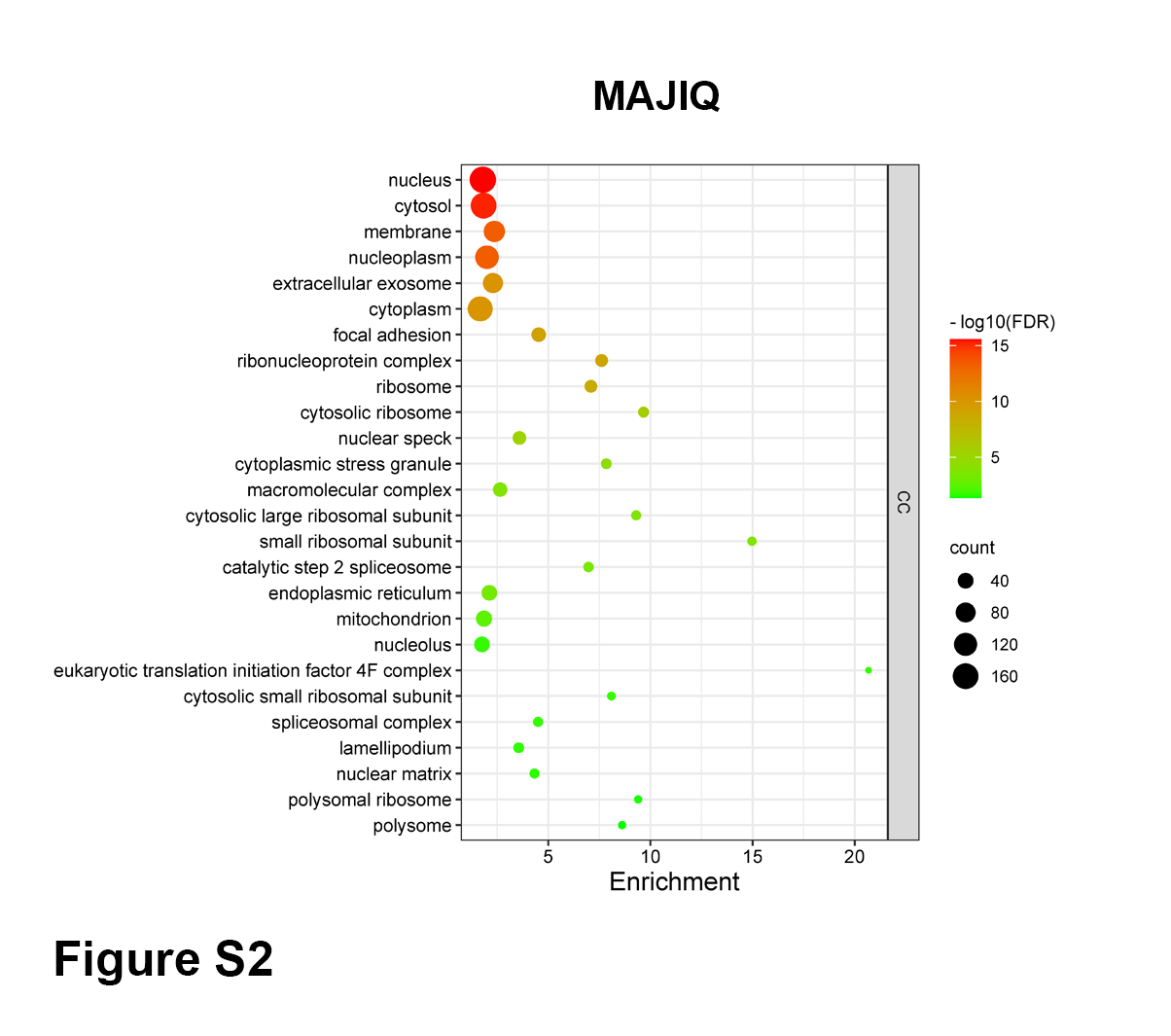

Supplement: S2 Fig — Gene Ontology analysis of genes predicted to contain alternative splicing events altered by ethanol by MAJIQ. CC, Cellular Component, FDR, False Discovery Rate. (TIF) [file pone.0284357.s002.tif]

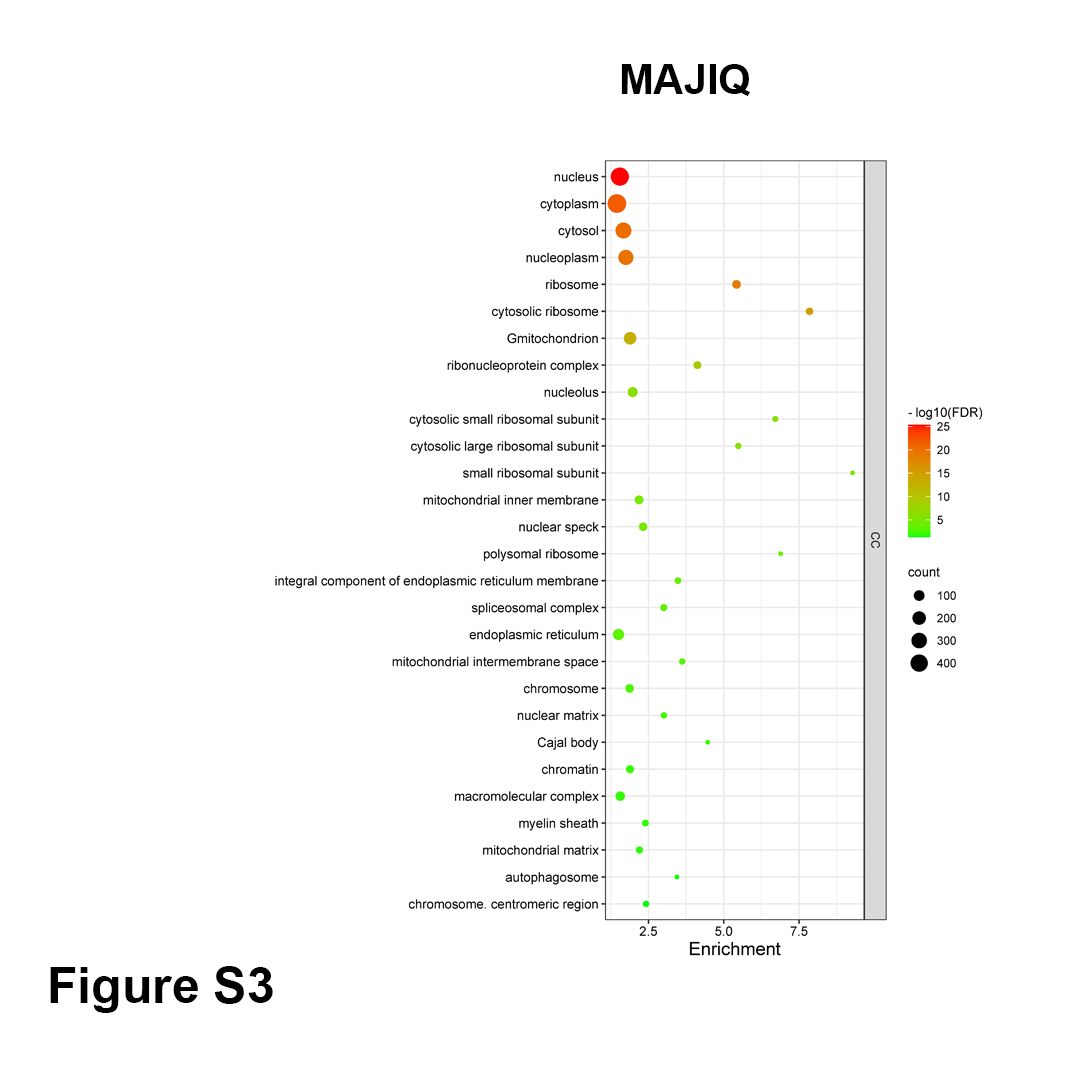

Supplement: S3 Fig — Gene Ontology analysis of genes predicted to contain alternative splicing events altered by ethanol by MAJIQ. CC, Cellular Component, FDR, False Discovery Rate. (TIF) [file pone.0284357.s003.tif]
