# Supplementary material for: Bioinformatic analysis predicts that ethanol exposure during early development causes alternative splicing alterations of genes involved in RNA post-transcriptional regulation
Source: PLoS One. 2023 Apr 13;18(4):e0284357. doi: 10.1371/journal.pone.0284357 (PMC10101408; doi:10.1371/journal.pone.0284357)
Supplement: S1 Appendix — Description of the execution of Alternative Splicing Software used in this work. (DOCX) [file pone.0284357.s009.docx]

**Additional File 6**

**Detailed Execution of Alternative Splicing Software**

**VAST-TOOLS**

VAST-TOOLS version 2.5.1 was used for the AS analysis. For each of the libraries the following set of commands were used.

Align module

- In the align command with the default parameters, each read is split into 50 nucleotides read groups. Each of these groups is aligned to the specified reference genome. Every unmapped read is aligned to a predefined splice junction library.

Code example

- ./VAST-TOOLS align sampleA_r1.fastq.gz sampleA_r2.fastq.gz –sp ‘species code’ –name ‘identifiable name’ –cores ‘N’ -o ‘output directory’
  - For each paired end sample.
  - If the sample is single end, a single file is required.
  - The species code in the –sp parameter is needed to correctly load the genomic data.
    - E.g mm10 for Mouse or hg38 for human

Combine module

- The combine command uses the processed files from the previous step and creates a table containing the necessary data for sample comparison.

Code example

- ./VAST-TOOLS combine -o ‘previously used output directory’ –sp ‘species code’ –cores ‘N’
  - The -o parameter needs the previously used output directory to fetch the aligned data.
  - The species code in the –sp parameter is needed to correctly load the genomic data.
    - E.g mm10 for Mouse or hg38 for human

Compare module

- Lastly, the compare command identifies alternative splicing events between two samples.

Code example

- ./VAST-TOOLS compare ‘INCLUSION TAB path’ -a ‘given name to samples A’ -b ‘given name to samples B’ -name_A ‘name to A group’ -name_B ‘name to B group’ –sp ‘species code’ –print_dPSI
  - The inclusion tab path is the first and mandatory parameter for the compare command. This is created in the previous step.
  - -a and -b are the identifiable name given to each sample in the align command.
  - -name_A and -name_B are descriptive names to each sample group.
  - The species code in the –sp parameter is needed to correctly load the genomic data.
    - E.g mm10 for Mouse or hg38 for human
  - –print_dPSI create a column with the difference of dPSI from group B and A

**rMATS**

rMATS version 4.1.2 was used for the AS analysis.

rMATS requires text files with the path of the samples to be analyzed. Each sample must be in FASTQ format and must be specified in the command if they are single or paired end. For each of the analyzed libraries the following command with the corresponding modifications was used.

Code example.

- rmats.py –s1 ‘sample A paths in text file’ –s2 ‘sample B paths in text file’ –gtf ‘gtf file’ –bi ‘STAR aligner index folder’ -t ‘single or paired’ –readLength ‘N’ –variable-read-length –od ‘output directory’ –tmp ‘temporary folder’
  - As stated, –s1 and –s2 parameters are for the samples to be analyzed. A text file with the path of the sample must be used. The paths must be comma separated and in a single line.
  - The annotation file in gtf format is required. The path to the file is specified in the –gtf parameter.
  - The –bi parameter is for the path where the output files for the STAR aligner are located.
  - The –readLength parameter allows to specify the length of the reads.
  - The –variable-read-length parameter allows reads that differs from the

–readLength parameter to be processed.

**MAJIQ**

MAJIQ version 2.4 was used for the AS analysis. A configuration file is required to run MAJIQ. This can be created from their website ([https://biociphers.bitbucket.io/MAJIQ-docs-academic/commandbuilder.html](https://biociphers.bitbucket.io/majiq-docs-academic/commandbuilder.html)) or using a base template like the following:

[info]

bamdirs=’directory of sorted BAM files’

genome=’code of the species to analyze. E.g mm10’

[experiments]

sampleA=file1.sorted.bam,file2.sorted.bam

sampleB=file1.sorted.bam,file2.sorted.bam

It is recommended to use the command builder tool This will allow the user to input the data and the information regarding the comparisons to be made and obtain a set of 3 commands lines involving the execution of MAJIQ.

Build module

- The build module in MAJIQ, uses sorted BAM files and annotations files (gff3) to determine known or novel AS or LSV.

Code example.

- MAJIQ build ‘genome gff3 file path’ -o ‘output directory’ -c ‘MAJIQ configuration file path’ –disable-denovo –disable-denovo-ir -j ‘N’
  - The first parameter corresponds to the path of the gff3 annotation file for the organism.
  - –disable-denovo and –disable—denovo-ir parameters tells MAJIQ to not quantify unannotated events.
  - –j parameter indicates the desired CPU threads to use.

Quantifier module

- The quantifier module in MAJIQ quantifies the relative abundance (PSI) of AS (LSV) events and changes in relative abundance (delta PSI) between conditions. This is used with the outputs of the previous module.

Code example.

- MAJIQ deltapsi -o ‘deltapsi output directory’ –j ‘N’ -grp1 ‘MAJIQ files for first group’ -grp2 ‘MAJIQ files for second group’ -n ‘name of the groups. E.g groupA groupB’
  - –o parameter dictates the output directory for the deltapsi files.
  - –j parameter indicates the desired CPU threads to use.
  - –grp1 and –grp2 are MAJIQ options for files from the previous step. These files end with ‘.MAJIQ’ extension.
  - -n desired name of the analyzed groups.

Viewer module

- The viewer module offers different modes for converting the quantified results to a human readable format. E.g., CSV or TSV

Code example

- voila tsv ‘splicegraph.sql path’ ‘.voila file from the previous module’ -f ‘output file name’ –show-all
  - The first parameter is the splicegraph.sql file created in the previous module.
  - The second parameter refers to the ‘.voila’ file created in the previous module.
  - The –f parameter takes an output path.
  - The –show-all parameter indicates that all the AS even registered will be written to the output file.

**MicroExonator**

MicroExonator version 1.0 was used for the AS analysis. A series of configurations files are required to run MicroExonator. This configuration files were created for each of the used libraries.

Local Files location.

As stated in the manual a ‘local_samples.tsv’ file must be created in the directory of MicroExonator to indicate where the files are located.

Example:

path sample

path/to/sample1.fastq.gz name_sample1

Configuration file.

MicroExonator requires a configuration file to run. The template to this file is the following. Taken directly from the manual.

Configuration File example:

Genome_fasta : /path/to/Genome.fa

Gene_anontation_bed12 : /path/to/ensembl.bed12

GT_AG_U2_5 : /path/to/GT_AG_U2_5.good.matrix

GT_AG_U2_3 : /path/to/GT_AG_U2_3.good.matrix

conservation_bigwig : /path/to/conservation.bw

working_directory : /path/to/MicroExonator/

ME_len : 30

Optimize_hard_drive : T

min_number_files_detected : 3

The Genome_fasta parameter indicates the path of the genome file to be analyzed. Gene_annotation_bed12 parameter indicates the path of the bed12 annotation file associated with the genome to be analyzed. GT_AG_U2_5 and GT_AG_U2_3 indicates the path of the files for the position weight matrices (PWM) associated with SpliceRack. These matrices indicate nucleotide frequencies in the intron-exon junction at each position (nucleotides). Files for human and mouse are available in the PWM folder of MicroExonator. If they are not available, an “NA” value can be used here, and the program will generate them from the annotated splice sites.

The conservation_bigwig is a *bigwig* file containing genome-wide conservation scores generated. For some species, they can be downloaded from UCSC genome browser. A “NA” can be used here if the file is not available.

The working_directory parameter indicates where the MicroExonator folder is. The ME_len parameter refers to the maximum length of a microexon and the *min_number_files_detected* parameter sets the minimum number of files where a microexon need to be found to consider it as a high confidence result.

For each of the analyzed libraries these files were created and placed under the root folder of MicroExonator.

Lastly, the following command to run MicroExonator was applied:

snakemake -s MicroExonator.smk -c 4 –use-conda –conda-frontend conda
